# Effectiveness of intraoperative endoscopic evaluation in aortic valve repair with valve-sparing aortic root replacement: a comparison of short- and mid-term results

**DOI:** 10.1093/icvts/ivae059

**Published:** 2024-04-03

**Authors:** Go Yamashita, Jota Nakano, Atsushi Sugaya, Jiro Sakai, Shingo Hirao, Tatsuhiko Komiya

**Affiliations:** Department of Cardiovascular Surgery, Kurashiki Central Hospital, Kurashiki, Japan; Department of Cardiovascular Surgery, Kurashiki Central Hospital, Kurashiki, Japan; Department of Cardiovascular Surgery, Kurashiki Central Hospital, Kurashiki, Japan; Department of Cardiovascular Surgery, Kurashiki Central Hospital, Kurashiki, Japan; Department of Cardiovascular Surgery, Kurashiki Central Hospital, Kurashiki, Japan; Department of Cardiovascular Surgery, Kurashiki Central Hospital, Kurashiki, Japan

**Keywords:** Aortic root replacement, Valve-sparing, Aortic valve insufficiency, Aortic valve repair

## Abstract

**OBJECTIVES:**

Valve-sparing aortic root replacement requires expertise to predict repair results and prevent secondary aortic clamping for valve repair or replacement secondary to aortic valve insufficiency. Thus, intraoperative evaluation of the aortic valve using diastolic pressure at the aortic root may be helpful. The goal of this retrospective study was to compare the early and mid-term results of aortic valve repair with those of valve-sparing aortic root replacement using intraoperative endoscopic evaluation.

**METHODS:**

We included 158 patients who underwent aortic valve repair with valve-sparing aortic root replacement at our hospital between December 2003 and January 2022. The patients were divided into a non-endoscopic evaluation group (group NE, n = 97; mean age 55 years) and an endoscopic evaluation group (group E, n = 61; mean age 51 years).

**RESULTS:**

The incidence of a second aortic clamping for aortic valve insufficiency was significantly greater in group NE (17.5%) than in group E (1.6%; *P* = 0.002). The presence of none or trivial aortic valve insufficiency on transthoracic echocardiography at discharge in group E (87.6%) was significantly lower than in group NE (98.4%; *P* = 0.017). No significant difference in the cumulative incidence of recurrence of moderate AI (P = 0.47), hospitalization for heart failure (P = 0.84) and reoperation (P = 0.25) between groups NE and E.

**CONCLUSIONS:**

Intraoperative endoscopic evaluation during aortic valve repair with valve-sparing aortic root replacement correlated with a lower incidence of second aortic clamping because of aortic valve insufficiency and effective aortic valve insufficiency control.

## Graphical Abstract

**Figure:**
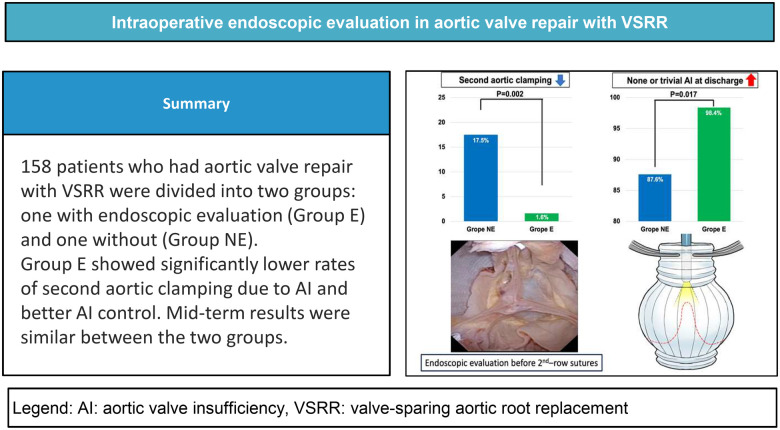

## INTRODUCTION

Valve-sparing aortic root replacement (VSRR) procedures, including aortic root remodelling and aortic valve reimplantation, were initially introduced as alternatives to the Bentall procedure by Yacoub and David nearly 30 years ago [1, 2]. The occurrence of future aortic valve insufficiency (AI) deterioration necessitating aortic valve reoperation ensues because of the intricacy of the procedure, coupled with the imperative requirement for a profound comprehension of the aortic root complex. A VSRR requires expertise to predict the repair outcome and avoid aortic reclamping in cases of subsequent AI. Reliable intraoperative indicators are essential for addressing the variability in anatomical features among diseased aortas, making the procedure reproducible, and eliminating reliance on the experience of a limited number of surgeons for making decisions. Intraoperative endoscopy is used to assess residual insufficiency and for the final assessment after aortic valve repair [3–5]. However, previous studies have described only endoscopic evaluations for final confirmation after second-row sutures. Therefore, only central plication or leaflet suspension was performed after the final evaluation. We introduced a new technique for aortic valve evaluation using an endoscope after first-row sutures before second-row sutures VSRR. Severe AI and more than mild regurgitation at discharge are predictors of late reoperation [6]. Patients undergoing root procedures are less likely to have higher-grade postoperative AI [7]. Consequently, our goal was to enhance the early and mid-term outcomes by incorporating intraoperative endoscopy into aortic valve repair with VSRR. However, the early and mid-term outcomes of these techniques remain unclear.

This study evaluated the effectiveness of the intraoperative endoscopic evaluation of aortic valve repair using VSRR.

## MATERIALS AND METHODS

### Ethical statement

This study adhered to the Declaration of Helsinki and its subsequent amendments. This retrospective observational study was conducted at a single centre. This study was approved by the institutional review board of Kurashiki Central Hospital (Approval No. 4128; Approval Date: 4 July 2023). The requirement for informed consent was waived because of the retrospective nature of the study.

### Study design

Patients who underwent aortic valve repair at our facility between December 2003 and January 2022 were included in this study. Exclusion criteria comprised cases without concomitant VSRR and those with AI graded as less than trivial. Additionally, participants underwent various concomitant procedures, such as coronary artery bypass grafting, other valve operations or thoracic aortic surgery. Consequently, the study cohort consisted of 2 groups: non-endoscopic evaluation (group NE) and endoscopic evaluation (group E). The allocation was based on whether the patients underwent endoscopic evaluation during aortic valve repair.

Patient characteristics and operative data were obtained from the medical records. Follow-up examination data, including survival and complications, were obtained via patient chart reviews at the outpatient clinic. Telephone follow-up was conducted for patients who did not attend the outpatient clinic. Eight cases underwent follow-up via telephone. All of them belonged to group NE, and among them, 3 cases had had transthoracic echocardiography (TTE). Follow-up was completed in all patients. Additionally, follow-up via letters was conducted for some patients. This meticulous approach ensures a comprehensive follow-up, and we want to explicitly state that follow-up regarding vital status was thorough and complete.

### Echocardiographic follow-up

The TTE was performed within 2 weeks postoperatively in all patients. After discharge, follow-up TTE was performed once a year.

### Classification of aortic insufficiency

In 2009, Boodhwani *et al.* [8] proposed a repair-oriented classification of AI, which we used for classification of AI in the present study. Surgeons determined the cause of AI by using repair-oriented classification in conjunction with echocardiography findings. Study patients before 2009 were retrospectively classified based on their operative records and preoperative echocardiography.

### Outcomes of interest

This study assessed various outcomes to compare early and mid-term results between the 2 groups. Early results encompassed second aortic clamping due to AI, none or trivial AI in TTE at discharge, in-hospital mortality rates and the rates of additional repair after endoscopic evaluation. Mid-term results included overall survival rates, freedom from cardiac death, cumulative incidence of hospitalization for heart failure, recurrence of moderate AI and reoperation rates.

### Surgical technique

**Figure 1:**
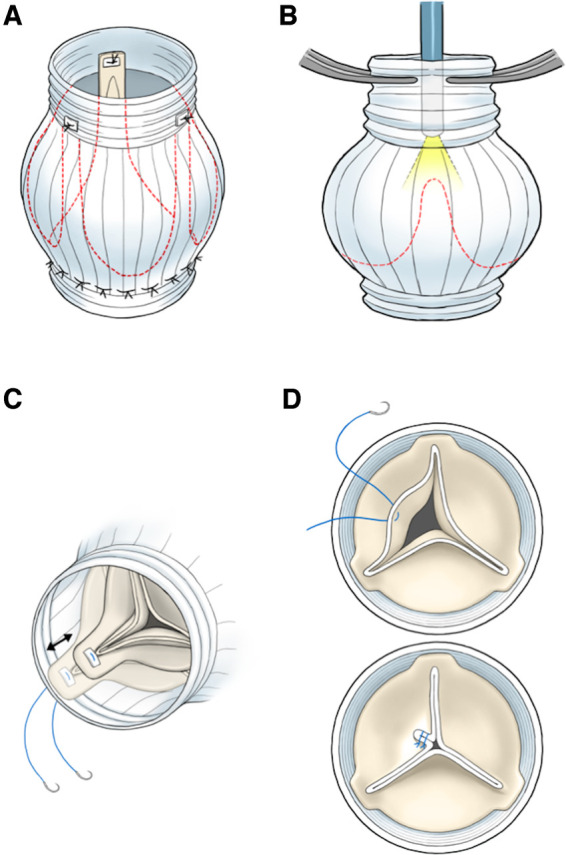
The first row is sewn, and each commissure is attached to the sinotubular junction of the vascular graft (**A**). The graft is clamped around the endoscope, and saline is infused into the graft via the endoscope. Aortic valve geometry is visualized, and the degree of valve coaptation is assessed (**B**). Surgical repair techniques include commissure position adjustment (**C**) and central plication (**D**). DLco: diffusing capacity for carbon monoxide; TFT: two-flight test.

Preoperatively, aortic valve geometry was assessed using three-dimensional, high-resolution multidetector computed tomography (CT), including measurement of the annular size and evaluation of the aortic cusps [9]. The aortic cusp with the highest effective height on the CT was used as the reference cusp. Surgery was performed via median sternotomy with standard cardiopulmonary bypass under mild hypothermia. We performed the VSRR technique described by David *et al.* [2]. After making a transverse incision 1 cm above the top of the commissure in the ascending aorta, the geometric height (GH), effective height, free margin length of each aortic cusp and commissure height were measured to confirm CT findings [10]. Effective height was measured using a caliper (Fehling Instruments, Karlstein am Main, Germany). Initially, aortic valve repair, such as central plication, was performed to adjust the length of the cusp margins. If there was excess tissue, some valve leaflets were excised, or plication was extended to the ridge with single stitches in order to reduce redundant leaflets and preserve their natural shapes. A Gelweave Valsalva graft (Terumo, Tokyo, Japan) was attached to the basal annulus using first-row sutures. Subsequently, each commissure was attached to the sinotubular junction of the graft using pledgeted 4–0 polypropylene sutures (Fig. 1A).

Intraoperative endoscopy was performed as follows: Before sewing the second-row sutures, endoscopic evaluation was performed by inserting a flexible endoscope (CYF-VHA, Olympus Medical Systems, Tokyo, Japan) into the vascular graft. To pressurize the aortic root, a three-way stopcock was affixed to the endoscope. We established 2 distinct channels: 1 for the infusion of saline and the other for pressure monitoring. The line designated for pressure monitoring is connected to the root vent pressure monitor for measurement. After clamping the vascular graft around the endoscope, saline was infused into the aortic root to mimic a diastolic aortic root pressure of up to 60 mmHg (Fig. 1B). Special attention was given to preventing aortic root deformation-induced aortic valve regurgitation by clamping the vascular graft around the endoscope approximately 5 cm above the sinotubular junction. Failure to achieve the target pressure indicated residual aortic insufficiency. The aortic root was also visualized using an endoscope to evaluate aortic valve coaptation. If the 2 non-reference cusps were higher or lower than the reference cusp, the commissural height between the non-reference cusps was adjusted (Fig. 1C). If 1 cusp exhibited residual prolapse, central plication was performed (Fig. 1D). The use of endoscopy before sewing the second-row sutures facilitates additional correction of the heights of the 2 coronary cusps by changing the commissure position. For large fenestrations, the free margin of the cusp was reinforced using a CV-7 Gore-Tex suture (W. L. Gore & Associates, Newark, DE, USA) to prevent late prolapse from re-fenestration string rupture. In the presence of restrictions and gaps at the commissure, fresh pericardial strips were used for commissuroplasty. If an endoscope is not used, it should be checked for prolapse based on the surgeon’s judgement. A second row of 4–0 polypropylene continuous sutures was initiated at the nadir of each sinus and sewn upward towards the top of the commissure to create a crescent shape. If needed, consider reevaluating the aortic valve endoscopically at this stage. Subsequently, the coronary arteries were implanted using 5–0 polypropylene continuous sutures, and distal anastomoses were performed. Finally, the presence and degree of AI were determined using intraoperative transoesophageal echocardiography supervised by an experienced anaesthesiologist or cardiologist. Residual AI was considered acceptable if the grade was less than mild, with a central jet. Even if the grade was mild, the aortic valve was repaired in the case of an eccentric jet (Video 1).

### Statistical analysis

The statistical analyses were performed using the SPSS Statistics 20.0 software package for Windows (IBM Corp., Armonk, NY, USA) and EZR (Saitama Medical Center, Jichi Medical University, Saitama, Japan), which is a graphical user interface for R (The R Foundation for Statistical Computing, Vienna, Austria). Continuous variables were calculated as the mean ± standard deviation (SD) or median [interquartile range (IQR)] and compared using the Student *t*-test or Mann–Whitney *U* test. Categorical variables were calculated as counts and percentages and compared using the χ^2^ or the Fisher’s exact test. Postoperative overall survival and freedom from cardiac death were estimated using the Kaplan–Meier method and compared using the log-rank test. The Gray test was applied to assess the cumulative incidence of hospitalization for heart failure, recurrence of moderate AI and reoperation between the 2 groups, along with removing the effects of the observed confounders of death before the events. A value of *P* < 0.05 was considered significant.

## RESULTS

Between December 2003 and January 2022, a total of 291 consecutive patients underwent aortic valve repair at our facility. Of these, we excluded 120 patients without VSRR and 13 patients with less than trivial AI. Therefore, the study cohort comprised 158 patients who underwent aortic valve repair with a VSRR, performed using the reimplantation technique. The patients were categorized as group NE (*n* = 97, 61.4%) and group E (*n* = 61, 38.6%).

### Preoperative patient characteristics

Analysis of the patient characteristics showed that severe preoperative AI and larger sinuses of Valsalva, as well as a larger ascending aorta diameter, were more prevalent in group E. The preoperative TTE parameters did not show statistically significant differences between groups NE and E. Patients with bicuspid aortic valves (BAV) were more prevalent in group E. Classification of AI in the study cohort is as follows: of the 158 patients, 61 had isolate type I AI; 27 had isolate type II AI; 67 had type I and II AI; 2 had type I and III AI; and 1 had type II and III AI. Type 1 AI was significantly more prevalent in group NE (*P* = 0.0001), whereas type 2 AI was more common in group E (*P* = 0.0001) (Table 1).

**Table 1:** Patient characteristics

Variables	Overall	Group NE	Group E	*P*-value
	n = 158	n = 97	n = 61	
Age (years)	57 (44-67)	58 (46-67)	54 (33-68)	0.33
Sex (male)	136 (86.1)	80 (82.5)	56 (91.8)	0.099
Body surface area (m²)	1.75 ± 0.17	1.74 ± 0.18	1.76 ± 0.15	0.64
Hypertension	117 (74.1)	74 (76.3)	43 (70.5)	0.42
Hyperlipidaemia	34 (21.5)	23 (23.7)	11 (18.0)	0.40
Smoking	79 (50.0)	48 (49.5)	31 (50.8)	0.87
Diabetes	2 (1.3)	1 (1.0)	1 (1.6)	0.74
CKD (eGFR < 45)	14 (8.9)	9 (9.3)	5 (8.2)	0.82
Previous stroke	7 (4.4)	6 (6.2)	1 (1.6)	0.18
Atrial fibrillation	14 (8.9)	12 (12.4)	2 (3.3)	0.081
Resternotomy	7 (4.4)	5 (5.2)	2 (3.3)	0.58
Marfan syndrome	10 (6.3)	9 (9.3)	1 (1.6)	0.090
Aortic valve morphology				
Unicuspid	1 (0.6)	1 (1.0)	0	0.43
Bicuspid	34 (21.5)	16 (16.5)	18 (29.5)	0.053
Tricuspid	123 (77.8)	80 (82.5)	43 (70.5)	0.077
Preoperative aortic valve insufficiency				
Mild	6 (3.8)	6 (6.2)	0	0.083
Moderate	31 (19.6)	23 (23.7)	8 (13.1)	0.15
Severe	121 (76.6)	68 (70.1)	53 (86.9)	0.020
Preoperative TTE parameters				
LAD, mm	39 (33-39)	39 (33-46)	39 (35-44)	0.55
LVDd, mm	58 (53-63)	58 (53-64)	58 (53-63)	0.87
LVDs, mm	41 (34-47)	40 (33-46)	41 (35-48)	0.42
LVEF, %	56 (50-61)	57 (50-61)	55 (50-62)	0.96
CT parameters				
Sinus of Valsalva, mm	45 (39-51)	46 (42-55)	40 (36-49)	0.0001
Ascending aorta, mm	39 (35-45)	42 (36-47)	37 (33-41)	0.001
Aortic arch, mm	31 (27-34)	31 (28-35)	29 (26-33)	0.011
Classification of aortic insufficiency				
Type I	61 (38.6)	49 (50.5)	12 (19.7)	0.0001
Type II	27 (17.1)	3 (3.1)	24 (39.4)	0.0001
Types I and II	67 (42.4)	43 (44.3)	24 (39.4)	0.62
Types I and III	2 (1.3)	2 (2.1)	0	0.52
Types II and III	1 (0.6)	0	1 (1.6)	0.39

Values are presented as n (%) or mean ± SD, or median (IQR);

CKD: chronic kidney disease; CT: computed tomography; eGFR: estimated glomerular filtration rate; LAD: left atrial dimension; LVDd: left ventricular end-diastolic dimension; LVDs: left ventricular internal dimension in systole; LVEF: left ventricular ejection fraction; TTE: transthoracic echocardiography.

### Early outcomes

Table 2 shows intraoperative data and early outcomes. The percentages of partial and total arch replacements as concomitant procedures were significantly greater in group NE (18.6%) compared to group E (1.0%; *P* = 0.001). Mean cardiopulmonary bypass and cross-clamp times did not differ between the 2 groups. The annulus diameter was smaller in group NE than in group E, and the GH of the right coronary cusp in the tricuspid aortic valve (TAV) and in the BAV, as well as the GH of the non-group NE, was larger in group E. The right free margin length in TAV was greater in group E, but there was no difference in the BAV. No significant differences were observed in postoperative TTE parameters between the 2 groups. The percentage of second aortic clamping because of AI in group NE (17.5%) was significantly greater than in group E (1.6%; *P* = 0.002). Only 1 patient in group E exhibited a mild AI with an eccentric jet observed in transoesophageal echocardiography after aortic declamping. The cusp had not originally prolapsed. After the second aortic clamping, central plication was performed on the prolapsed cusp. The percentages of none or trivial AI in TTE at discharge were significantly lower in group E (98.4%) than in group NE (87.6%; *P* = 0.017). Only 1 patient in group E had a mild AI with a central jet in TTE at discharge.

**Table 2:** Intraoperative variables and early results

Variables	Group NE	Group E	*P*-value
	n = 97	n = 61	
Emergency operation	3 (3.1)	2 (3.3)	0.95
Concomitant procedures	54 (55.7)	24 (39.3)	0.051
CABG	2 (2.1)	3 (4.9)	0.32
MVP	11 (11.3)	7 (11.5)	0.98
TAP	2 (2.1)	2 (3.3)	0.64
MAZE	10(10.3)	2 (3.3)	0.10
Hemiarch replacement	13 (13.4)	9 (14.8)	0.81
Partial or total arch replacement	18 (18.6)	1 (1.6)	0.001
Mean CPB time (min)	253 (213–290)	228 (207–273)	0.097
Mean cross-clamp time (min)	198 (169–219)	183 (168–220)	0.40
Valsalva graft size	28 (26–28)	26 (26–28)	0.30
Intraoperative measurement			
Annulus diameter (mm)	26 (25–28)	27 (26–28)	0.011
GH of right coronary cusp in TAV (mm)	19.6 ± 2.7	18.4 ± 2.3	0.019
GH of non-coronary cusp in TAV (mm)	20.2 ± 2.9	19.4 ± 2.2	0.11
GH of left coronary cusp in TAV (mm)	19.0 ± 2.4	18.3 ± 1.9	0.11
GH of right coronary cusp in BAV (mm)	19.5 ± 3.4	17.5 ± 1.9	0.04
GH of non-coronary cusp in BAV (mm)	23.4 ± 1.8	19.7 ± 2.5	0.0001
GH of left coronary cusp in BAV (mm)	19.2 ± 3.6	18.2 ± 2.5	0.37
Right free margin length in TAV (mm)	41.2 ± 7.1	45.0 ± 6.9	0.006
Non free margin length in TAV (mm)	38.8 ± 6.2	40.6 ± 7.0	0.145
Left free margin length in TAV (mm)	37.6 ± 6.3	40.0 ± 6.3	0.051
Non-fused cusp length in BAV (mm)	41.6 ± 5.2	41.5 ± 7.5	0.96
Fused cusp length in BAV (mm)	48.6 ± 10.6	49.8 ± 8.3	0.70
Postoperative TTE parameters			
LVDd, mm	49 (45–53)	49 (46–53)	0.29
LVDs, mm	35 (30–41)	37 (32–41)	0.26
LVEF, %	50 (44–56)	49 (40–52)	0.09
Second aortic clamping due to AI	17 (17.5)	1 (1.6)	0.002
None or trivial AI at discharge	85 (87.6)	60 (98.4)	0.017
In-hospital death	1 (1.0)	0	0.43
Additional repair after endoscopic evaluation		40 (65.6)	

Values are presented as n (%) or mean ± SD, or median (IQR).

AI: aortic valve insufficiency; BAV: bicuspid aortic valve; CABG: coronary artery bypass grafting; CPB: cardiopulmonary bypass; GH: geometric height; LVDd: left ventricular end-diastolic dimension; LVDs: left ventricular internal dimension in systole; LVEF: left ventricular ejection fraction; MVP: mitral valve plasty; TAP: tricuspid annuloplasty; TAV: tricuspid aortic valve; and TTE: transthoracic echocardiography.

In-hospital mortality did not differ significantly between groups NE (1.0%) and E (0%; *P* = 0.426). One in-hospital death occurred postoperatively: a case of aortic valve repair and VSRR combined with ascending and total aortic arch replacement due to intestinal ischaemia caused by the bending of a conventional elephant trunk inserted at the distal anastomosis. The cause of death was gastrointestinal haemorrhage following intestinal ischaemia. Re-exploration of the mediastinum for bleeding or tamponade was performed in 5 patients (3.2%). All re-explorations were performed in group NE.

Table 3 shows the details of the additional repair following endoscopic evaluation. Additional procedures were performed in 40 of 61 endoscopic cases (65.6%), with 9 cases involving multiple procedures, resulting in 51 procedures. After endoscopic evaluation, the most commonly performed additional procedure was aortic cusp plication, followed by commissural height readjustment (Fig. 1C and 1D). One case involved reinforcement of the free margin with Gore-Tex for residual perforation; the other involved reducing the first-row stitch by 1 stitch after determining that the valve leaflet was slightly pulled by it, and 1 required adding a Cabrol stitch.

**Table 3:** Additional repairs after endoscopic evaluation

Variables	N = 51
	(Patients n = 40)
Central plication	30 (58.8)
Readjustment of a commissural height	18 (35.2)
Free margin reinforcement with Gore-Tex	1 (2.0)
Reduced number of first-row stitches	1 (2.0)
Cabrol stitch	1 (2.0)

Values are presented as n (%).

### Mid-term outcomes

**Figure 2:**
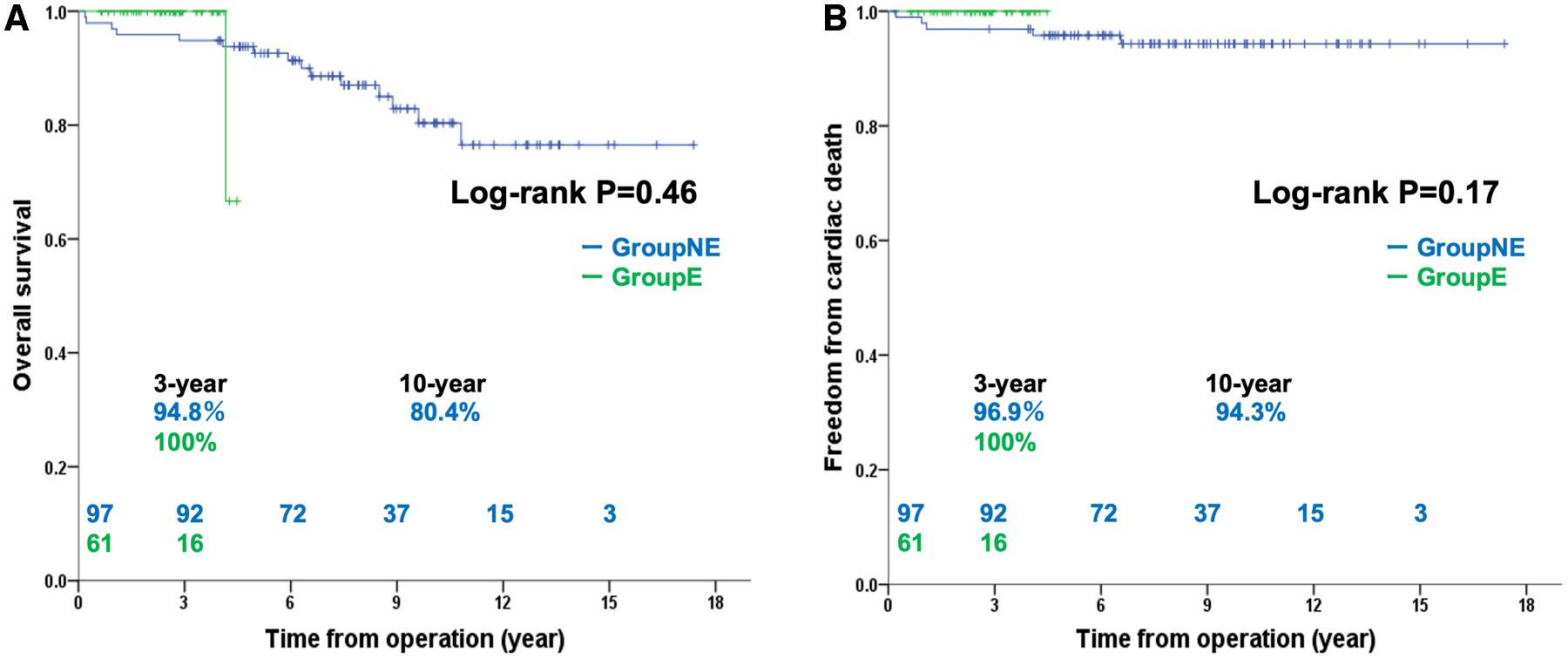
(**A**) Overall survival rates between groups NE and E. (**B**) Freedom from cardiac death between groups NE and E. DLco: diffusing capacity for carbon monoxide; TFT: two-flight test.

The median follow-up period was 5.1 years (IQR: 2.7–8.9 years, maximum 17.4 years). In group NE, the median follow-up was 7.9 years (IQR: 5.8–10, maximum 17.4 years), whereas in group E, the median follow-up was 2.7 years (IQR: 1.7–3.3, maximum 4.5 years). Group NE experienced 15 (15.5%) all-cause deaths; group E experienced only 1 (1.6%) death. No significant difference in overall survival rates was found between groups NE (94.8% at 3 years, 80.4% at 10 years) and E (100% at 3 years; *P* = 0.46) (Fig. 2A). No significant difference in freedom from cardiac death was found between groups NE (96.9% at 3 years, 94.3% at 10 years) and E (100% at 3 years; *P* = 0.17) (Fig. 2B). Group NE had 5 (5.2%) cardiac deaths; group E had no fatalities. The patients’ causes of death included heart failure (*n* = 2; 1.1 and 6.5 years postoperatively), ventricular fibrillation (*n* = 2; 0.3 and 1.0 years postoperatively), and acute myocardial infarction (*n* = 1; 4.1 years postoperatively).

**Figure 3.**
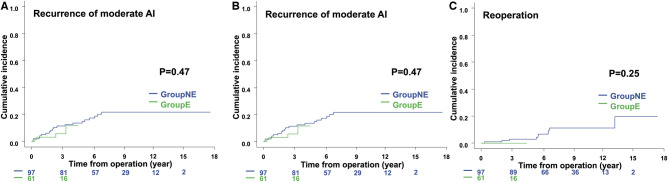
(**A**) The cumulative incidence of the recurrence of moderate aortic insufficiency between groups NE and E. (**B**) The cumulative incidence of hospitalization for heart failure between groups NE and E. (**C**) The cumulative incidence of reoperation between groups NE and E. DLco: diffusing capacity for carbon monoxide; TFT: two-flight test.

No significant difference in the incidence of recurrence of moderate AI was found between groups NE and E (*P* = 0.47; Fig. 3A). Group NE included 22 patients (22.7%) with more than moderate AI; group E included 4 patients (6.6%). No significant difference was found in the cumulative incidence of hospitalization for heart failure between groups NE and E (*P* = 0.84; Fig. 3B). Nine patients (9.7%) in group NE and 1 patient (1.6%) in group E were hospitalized. No significant difference was found in the cumulative incidence of reoperation between groups NE and E (*P* = 0.25; Fig. 3C). Ten patients (10.3%) in group NE underwent aortic valve reoperation; group E did not undergo any aortic valve reoperations. Among the patients who underwent reoperation, the aortic valve morphology was BAV in 3 cases and TAV in 7 cases, with 9 cases of AI and 1 case of AI with aortic valve stenosis. An autologous pericardial patch was used in 2 cases. Reoperations included aortic valve replacement in 8 cases and aortic valve repair in 2 cases. No patients had reoperation for infective endocarditis.

## DISCUSSION

Our investigation revealed that (i) intraoperative endoscopic evaluation during aortic valve repair with VSRR correlated significantly with a decreased second aortic clamping due to AI and demonstrated excellent control of aortic valve regurgitation. (ii) No significant differences were found in the mid-term results based on intraoperative endoscopic evaluation.

VSRR has been performed for more than 2 decades, yielding excellent long-term results [11–13). However, precise surgical techniques and an understanding of the anatomy of the aortic valve are required. One method for aortic valve evaluation is intraoperative endoscopy, which aids in assessing the need for additional repair. Arbakhani *et al.* has also elaborated on the value of this approach for aortic valve plasty. The postoperative residual AI grade was found to correlate with the leakage measured by their intraoperative device and was validated for use during AVP procedures [14]. However, previous studies described only endoscopic evaluations for final confirmation after second-row suturing [3, 4, 15]. If residual insufficiency remains after the second-row sutures, the commissural heights cannot be adjusted, even though aortic valve repair can be performed. Therefore, we propose performing an endoscopic evaluation after the first-row sutures and before the second-row sutures. Because endoscopy can reveal residual insufficiency and misalignment of the cusps, surgeons can adjust the commissural height. Readjustment of commissural height can help align the 2 cusps simultaneously (Fig. 1D). Pressure monitoring is necessary to prevent damage to the cusps. This method is more suitable for aortic valve reimplantation than remodelling [16] because the commissure position is adjustable. It is also effective for AI without aortic valve prolapse because the heights of all cusps are rarely the same. Even though the tissue is soft and easily stretchable, measurements are unreliable, leading to residual prolapse and poor coaptation. Therefore, intraoperative endoscopy helps make fine adjustments.

Several good long-term results of aortic valve reimplantation have been reported [13, 17, 18]. One reason for these good results is that VSRR in the reimplantation technique stabilizes the functional aortic annulus and is associated with improved outcomes in patients undergoing TAV and BAV [19, 20] reimplantation. David *et al.* prospectively followed 333 consecutive patients who underwent aortic valve reimplantation between 1989 and 2012 with a mean clinical follow-up of 10.3 (SD: 6.8) years [17]. The freedom from aortic valve reoperation at 15 and 20 years was 96.9 (SD: 1.3)%, and freedom from moderate to severe AI at 15 and 20 years was 96.2 (SD: 1.0)%. This study had better results than ours, but only 43.2% of patients had moderate and severe AI compared to 96.2% in our study. David *et al.* did not use patch augmentation of the cusps in this series, and most patients had normal or near-normal cusps at the time of the operation. David *et al.* had the most skilled aortic valve reimplantation surgeon; however, their cohort differs from our target patients. Chirichilli *et al.* showed that moderate and severe AI occurred in 33.6% fewer of their patients than we saw in the present study [13]. Reflecting this complexity, type 2 AI accounted for 61.4% (97 cases) of the total cohort. The rate was particularly high in group E (80.3%).

In our retrospective study of patients with preoperative AI, the cumulative incidence of recurrence of moderate AI was not significantly different between the 2 groups (*P* = 0.47). Deas *et al.* reported that 111 patients (65 with TAV and 46 with BAV) with mild aortic insufficiency underwent VSRR between 2005 and 2019 [19]. The median follow-up period was 49 months (IQR, 12–93). In this group, freedom from recurrence of mild AI at 3, 5 and 10 years was 96.2%, 93.6% and 87% for BAV and 94.5%, 90.7% and 92% for TAV, respectively. Although this study also showed good long-term results, the inclusion criterion was normal aortic valve cusps. To our knowledge, the mid-term results of VSRR with a high frequency of above-moderate AI have not yet been reported. Long-term outcomes could not be compared in this study because of the shorter observation period in group E (median follow-up period: 2.7 years, IQR: 1.7–3.3, maximum 4.5 years) compared to group NE (median follow-up period: 7.9 years, I: 5.8–10, maximum 17.4 years), mainly because the endoscopic evaluation was introduced in 2018. However, this report may indicate aortic valve repair with VSRR in patients with moderate and severe AI. Because the endoscopic results remain short to mid-term reports, further long-term follow-ups may indicate improved results beyond the mid-term with endoscopic evaluation.

### Limitations

This study has several limitations. It was limited by its relatively small sample size and its retrospective and observational design. Given that the data were retrospectively obtained from medical records and telephone calls, evaluating the validity of the retrieved information presented a challenge. Nevertheless, we undertook measures to address this concern and bolster the reliability of our findings. Our hospital follows a protocol that mandates the documentation of complications and related information in a dedicated database during outpatient visits. Furthermore, throughout the data collection phase, multiple individuals meticulously scrutinized the data to ensure accuracy. The relatively small sample size in our study presented challenges in implementing advanced statistical methods to account for potential confounding factors, such as multivariate analysis or propensity score matching. Although endoscopic evaluation to mimic diastole is a simple and easy-to-use method, it does not provide objective measurements of the coaptation depth or the equality of coaptation line lengths. Importantly, the patient selection for VSRR lacked precise criteria, and the majority of operations (*n* = 157, 99.4%) were conducted by an experienced surgeon, leading to a potential learning curve bias, especially considering that endoscopic analysis was carried out in the latter part of the cohort. To address these limitations, a larger patient cohort is necessary for a more detailed and accurate assessment of long-term results.

## CONCLUSION

Intraoperative endoscopic evaluation of aortic valve repair with VSRR is associated with a lower occurrence of a second aortic clamping due to AI and excellent control of aortic valve regurgitation. No significant differences were found in mid-term results using endoscopic evaluation. Further research with a larger cohort is warranted to address unadjusted confounders due to the small sample size.

## Data Availability

The data underlying this article will be shared on reasonable request to the corresponding author.

## Notes

Presented at the 37th Annual Meeting of the European Association for Cardio-Thoracic Surgery, Vienna, Austria, 4–7 October 2023.

## ABBREVIATIONS

AI: Aortic valve insufficiency
BAV: Bicuspid aortic valve
CT: Computed tomography
GH: Geometric height
IQR: Interquartile range
SD: Standard deviation
TAV: Tricuspid aortic valve
TTE: Transthoracic echocardiography
VSRR: Valve-sparing aortic root replacement
